# Glyphosate Herbicide: Reproductive Outcomes and Multigenerational Effects

**DOI:** 10.3389/fendo.2021.672532

**Published:** 2021-07-07

**Authors:** María Mercedes Milesi, Virginia Lorenz, Milena Durando, María Florencia Rossetti, Jorgelina Varayoud

**Affiliations:** ^1^ Instituto de Salud y Ambiente del Litoral (ISAL), Facultad de Bioquímica y Ciencias Biológicas, Universidad Nacional del Litoral (UNL) - Consejo Nacional de Investigaciones Científicas y Técnicas (CONICET), Santa Fe, Argentina; ^2^ Cátedra de Fisiología Humana, Facultad de Bioquímica y Ciencias Biológicas, Universidad Nacional del Litoral (UNL), Santa Fe, Argentina; ^3^ Departamento de Bioquímica Clínica y Cuantitativa, Facultad de Bioquímica y Ciencias Biológicas, Universidad Nacional del Litoral (UNL), Santa Fe, Argentina

**Keywords:** glyphosate, glyphosate-based herbicides, estrogenic effects, female fertility, adverse reproductive outcomes, implantation failures, maternal exposure, multigenerational effects

## Abstract

Glyphosate base herbicides (GBHs) are the most widely applied pesticides in the world and are mainly used in association with GBH-tolerant crop varieties. Indiscriminate and negligent use of GBHs has promoted the emergence of glyphosate resistant weeds, and consequently the rise in the use of these herbicides. Glyphosate, the active ingredient of all GBHs, is combined with other chemicals known as co-formulants that enhance the herbicide action. Nowadays, the safety of glyphosate and its formulations remain to be a controversial issue, as evidence is not conclusive whether the adverse effects are caused by GBH or glyphosate, and little is known about the contribution of co-formulants to the toxicity of herbicides. Currently, alarmingly increased levels of glyphosate have been detected in different environmental matrixes and in foodstuff, becoming an issue of social concern. Some *in vitro* and *in vivo* studies have shown that glyphosate and its formulations exhibit estrogen-like properties, and growing evidence has indicated they may disrupt normal endocrine function, with adverse consequences for reproductive health. Moreover, multigenerational effects have been reported and epigenetic mechanisms have been proved to be involved in the alterations induced by the herbicide. In this review, we provide an overview of: *i)* the routes and levels of human exposure to GBHs, *ii*) the potential estrogenic effects of glyphosate and GBHs in cell culture and animal models, *iii*) their long-term effects on female fertility and mechanisms of action, and i*v)* the consequences on health of successive generations.

## Introduction

Since the genetic engineering revolution applied to agricultural crops two decades ago, area destined to genetic modified crops tolerant to herbicides have dramatically extended around the world, representing almost 90% of the farmlands. Most of these areas have been covered by glyphosate-tolerant crops, which triggered an increase in the use of glyphosate-based herbicides (GBHs) (1, 2). In addition to that, the annual global production of GBH formulations was estimated to be about one million tons (3). Particularly, in Argentina, one of the main producing countries of glyphosate tolerant crops (4), the marketing of GBHs reached nearly 200,000 tons in 2012, which represented 80% of total commercialized herbicides (5). Moreover, GBHs are non-selective, systemic, post-emergence herbicides (6) used for weed control on woody and herbaceous crops, but also for the maintenance of areas not used for agriculture, such as public, industrial spaces, in embankments, roadsides, homes and gardens (7). Therefore, due to the massive use and the variety of applications, GBHs have become the most widely applied pesticides worldwide (2).

Glyphosate [N-(phosphonomethyl) glycine], the active principle of GBHs, targets the enzyme 5-enolpyruvylshikimate-3-phosphate synthase (EPSPS) of the shikimate pathway, a metabolic route for the biosynthesis of aromatic compounds in plants and microorganisms (8). The EPSPS converts shikimate-3-phosphate to 5-enolpyruvylshikimate-3-phosphate, leading then to the biosynthesis of chorismate. Chorismate is the precursor of tyrosine, phenylalanine and tryptophan, but also of secondary metabolites, including ubiquinone, menaquinone, lignans, tannins, and flavonoids (9).

Glyphosate and its main metabolite, the aminomethylphosphonic acid (AMPA), are considered as ‘least toxic’ (category IV) substrates for mammals by the regulatory agencies, based on toxicity data, low environmental persistence, and the mechanism of action which is supposed to be confined to soil microbial and plants. Despite their apparent safety, recent evidence indicates that the mammal gut microbiome could be influenced by glyphosate. In this sense, some studies investigated the effects of glyphosate in microbiome of rats (10–12), cows (13) and pigs (14). Importantly, recent findings from Mesnage et al. (15) demonstrated that glyphosate treatment resulted in higher levels of intermediates of the shikimate pathway in the ceca, suggesting inhibition of EPSPS in the rat cecum microbiome. Although the rat gut microbiome is different from that of humans, epidemiological studies will be necessary to analyze whether environmental doses of glyphosate could disrupt gut microbiome metabolism. On the other hand, evidence has shown long half-lives depending on soil properties and environmental conditions, and harmful effect on human, animal and ecosystem health (16). This scenario is further aggravated whether one take into account the indiscriminate and negligent use of GBHs, which promoted the emergence of glyphosate resistant weeds, and consequently, the rise in the use of these herbicides and others to control them (17, 18). In this context, alarming levels of contamination exist, which is evidence by the detection of glyphosate and AMPA in different environmental matrixes such as surface waters (19), groundwaters and open-reservoir tank waters (20), rainwaters and soil (21), dust (22) and air (23). Biomonitoring studies have also suggested that humans are likely to be exposed to glyphosate through drinking water (24) and foodstuff such as soy-based infant formula (25) and soy sauce (26). Environmental and food contamination by glyphosate have raised concern about the health effects on humans and non-target organisms because of potential chronic exposure with social consequences.

GBH formulations are constituted by other chemicals in addition to glyphosate, named as “inert ingredients” which enhance herbicide action by facilitating penetration into plant tissues. The chemical composition of these formulations is considered as confidential business information, so that, to date, little is known about the contribution of co-formulants to the toxicity of herbicides (27). Nowadays, the safety of glyphosate and its formulations remain to be a controversial issue, as evidence is not conclusive whether the adverse effects are caused by GBH or glyphosate alone. While some studies have shown that formulations are more toxic than the active ingredient (28–30), other works found similar adverse effects when exposed to glyphosate or GBH (31), or even stronger toxic effects of glyphosate compared to GBH in the short term (32). Probably, this controversy arises because of differences in composition between the brand names of GBH formulations, which highlight the importance of conducting not only comparative studies between the GBHs and the active principle, but also assaying a wide range of commercial formulations.

Growing evidence from both *in vitro* and *in vivo* studies has indicated that glyphosate and its commercial formulations may disrupt normal endocrine function, with effects on reproductive development and detrimental consequences on reproduction. The observed effects were diverse and some include hormonal imbalance (31–33), proliferation/mitotic index alterations (33, 34), deregulation of proteins and genes involved in endocrine pathways (31, 34, 35), oxidative stress (36), as well as, epigenetic disruption such as, alteration in DNA methylation levels and/or histone post-translational modifications (PTMs) (37). Importantly, some results indicate that glyphosate appears to cause multigenerational effects, which might be transmitted transgenerationally to future generations (38, 39).

Despite these findings, the Endocrine Disruptor Screening Program lead by the United States Environmental Protection Agency (40) and later, the European Food Safety Authority (EFSA) (41) resolved that there was not sufficient evidence to support endocrine disrupting properties of glyphosate, turning this issue in another topic of conflict and scientific debate.

This review provides an overview of the routes of human exposure, the potential estrogenic effects of glyphosate and GBHs, their long-term effects on female fertility and mechanisms of action, and finally, the consequences on health of successive generations.

## Routes and Levels of Human Exposure to GBHs

Current studies on pesticides indicate that human exposure can occur through occupational use or proximity to areas with agricultural activity. However with the introduction of transgenic crops tolerant to agrochemicals, pesticide uses increased significantly and diet became one of the main sources of exposure to them (42–44). In transgenic glyphosate-tolerant crops, glyphosate can be applied to the crop (post-emergence) to remove emerged weeds without crop damage. Two strategies have been successful in introducing glyphosate tolerance: a) expression of an insensitive form of the target enzyme, and b) detoxification of the glyphosate molecule. The first one is used in existing commercial glyphosate-tolerant crops: the employment of a microbial or a mutated form of EPSPS that is not inhibited by glyphosate. The disadvantage of this approach is that glyphosate could remain and be accumulated in the plant (45).

In Argentina, glyphosate levels were found in the order of mg/kg in transgenic soybean seeds and plants in farmlands (46, 47) (**Table 1**). In 7 out of 11 samples of soybeans, the maximum residue limits (MRLs) for soybean of 20 mg/kg established by the European Commission were exceeded, as reported a non-peer reviewed study conducted by Testbiotech, a German financed independent organization (47). Later studies have shown that a variety of cereals grains (barley, oat, rye, wheat) (48), pulses (50) and related food (25, 49) reported detectable levels of glyphosate and/or AMPA (**Table 1**), however they agree that those residues are below the current MRLs and the acceptable daily intake (ADI) corresponding to 0.5 mg/kg bw/day according to the EFSA (55). A worrying finding was the determination of contaminated soy-based formulas for infants from different commercial brands in a study performed in Brazil during 2012-2017. The residues of glyphosate were from 0.03 mg/kg to 1.08 mg/kg and of AMPA were from 0.02 mg/kg to 0.17 mg/kg (25). According to Brazilian regulation, MRLs for this kind of product should be in line with the soybean raw material, which is 10 mg/kg (56); while the European Community establishes lower MRLs in cereal-based food and baby food for infants and young children of 0.01 mg/kg total (57). This example denotes great discrepancies between government institutions in relation to tolerable residues values in food. Data on the ingestion of GBH residues through foodstuffs still needs to be collected to continue monitoring the safety of various foods.

**Table 1:** Glyphosate and AMPA concentrations in food, water and air.

Matrixes	Area	Lab Method	LOD/LOQ	Glyphosate levels	AMPA levels	Reference
**Cereal, pulses and related food**						
Barley	Denmark	LC-MS/MS	LOD: 0.2 mg/kg	<0.45 mg/kg	NR	(48)
Oat				<0.08 mg/kg		
Rye				<0.04 mg/kg		
Wheat				<0.13 mg/kg		
Bread	Switzerland	LC-MS/MS	LOQ: 0.0005–0.0025 mg/kg	<0.001–0.0458 mg/kg	<0.0025 mg/kg	(49)
Breakfast cereal				<0.001–0.291 mg/kg	<0.0025–0.010 mg/kg	
GM Soybean Leaves/stems	Argentina	HPLC	LOD: 0.2 mg/kg,	1.9 - 4.4 mg/kg	1.9 - 4.4 mg/kg	(46)
GM Soybean Grains			LOQ: 0.15 mg/kg	0.1 - 1.8 mg/kg		
Whole GM soybeans	USA	HPLC–FLD	NR	0.4–8.8 mg/kg	0.7–10.0 mg/kg	(50)
GM soybeans grains	Argentina	HPLC	NR	5.3–25.8 mg/kg	<33.8 mg/kg	(47)
Soy-based infant formula	Brazil	HPLC–FLD	LOQ: 0.02 mg/kg	0.0.3–1.08 mg/kg	0.02–0.17 mg/kg	(25)
**Water bodies (rivers, lakes, tributaries)**						
Surface water	Canada	UHPLC- HRMS	LOD 0.002 µg/l (Glyphosate) and 0.01 µg/l (AMPA)	<0.002–3 µg/l	<0.010-0.656 µg/l	(19)
Water	Argentina	HPLC-MS	NR	<0.3 µg/l	<0.7 µg/l	(51)
SPM				<0.04 µg/l	<0.21 µg/l	
Sediments				<3.004 mg/kg	<5.374 mg/kg	
Water	Argentina	HPLC-MS	NR	1.25-4.52 µg/l	0.77-0.9 µg/l	(52)
SPM				0.04-0.13 µg/l	0.06 µg/l	
Sediments				0.004-0.020 mg/kg	0.012-0.032 mg/kg	
Groundwater	Argentina	UHPLC-MS/MS	LOD: 0.2 µg/l LOQ: 0.6 µg/l (Glyphosate) and 0.2 µg/l (AMPA)	0.6-11.3 µg/l	0.2-6.5 µg/l	(20)
Drinking tank water				0.6-21.2 µg/l	0.2-4.2 µg/l	
Surface water	Sri Lanka	LC-MS	LOD: 0.1 µg/l	28-45 µg/l	<1 µg/l	(53)
Groundwater			LOQ: 0.1 µg/l	1-4 µg/l	<11 µg/l	
Sediments				0.085-1.011 mg/kg	<0.015 mg/kg	
Surface water	Mexico	ELISA	LOD: 0.05 µg/l	0.33-4.36 µg/l	NR	(54)
Groundwater				0.26-3.17 µg/l		
Bottle water				<0.05 µg/l		
Runoff water				0.11-0.17 µg/l		
Groundwater	Mexico	ELISA	LOD: 0.05 µg/l	0.44-1.41 µg/l	NR	(24)
Drinking water			LOQ: 0.13 µg/l	0.35-0.65 µg/l		
Rain water	Argentina	UHPLC-MS/MS	LOD: 0.25 µg/l	0.5 - 2 µg/l	1.5-7 µg/l	(9)
Rain water	Argentina	HPLC-MS	LOD: 0.5 µg/l LOQ: 1 µg/l	0.5-67.28 µg/l	0.75- 7.91 µg/l	(51)
**Air**						
Respirable dust of agricultural soil	Argentina	UPLC	LOD: 0.36 µg/l (Glyphosate) and 0.41 µg/l (AMPA) LOQ 1.19 µg/l (Glyphosate) and 1.6 µg/l (AMPA)	11.0-19.5 µg/l	520-750 µg/l	(52)

ELISA, enzyme-linked immunosorbent assay; FLD, fluorescence detector; HPLC, High Performance Liquid Chromatography; HRMS, high resolution mass spectrometry; LC, liquid chromatography; LOD, limit of determination; LOQ, limit of quantification; MS/MS, tandem mass spectrometry; MS, mass spectrometry; NR, not reported; SPM, suspended particulate matter; UHPLC, Ultra High Performance Liquid Chromatography; UPLC, Ultra Performance Liquid Chromatography.

In regions with high agricultural activity, glyphosate and its mains metabolite (AMPA) have been detected with high frequency in sediments and surface waters from rivers and lakes (19, 51, 52); and also, in groundwaters and open-reservoir tank waters (20, 53, 54) (**Table 1**). Water resources can result contaminated by glyphosate and AMPA mainly due to the contribution of rainfall and surface runoff and to a lesser extent, by subsurface leaching or accidental spills, which might affect the ground and surface water deteriorating the drinking water quality as reported by Rendón-von Osten & Dzul-Caamal (24) in Mexico (**Table 1**). The presence of glyphosate in groundwater and bottled drinking water in this work (highest mean level 0.65 µg/l) indicated the excessive use of glyphosate in the agricultural communities studied (24). Regarding drinking water quality standards, each country has its own requirement of glyphosate residues. For instance, the maximum contaminant level for glyphosate is 280 µg/l in Canada and Argentina, 1000 µg/l in Australia, 700 µg/l in the US (58) and 0.1 µg/l in European Union (59). The differences between countries are due to the legislation on the use of genetic modified organisms and the number of hectares where this herbicide is applied (60). It should be noted that in a study performed in Argentina, glyphosate and AMPA have been identified at concentrations above the maximum permitted by the European Union in most of the rainwater samples analyzed (21). This information evidence the ubiquitous presence of glyphosate in water bodies and the potential exposure to glyphosate residues through dermal contact or ingestion.

Also, the presence of glyphosate in water resources might impact to a wide-range of non-target aquatic organisms. The toxic effects of glyphosate and GBH formulations have been extensively reported on algae, invertebrates and vertebrates from marine and river environments as evidenced by reviews on this topic (61, 62). It should be noted that several works revised by Matozzo´s review (62) showed that the herbicide can affect biological responses after chronic exposure in marine species at low concentrations (in the order of magnitude of µg/l). In addition, an interesting work which carried out a hazard assessment for glyphosate in a river from Argentina indicated that aquatic and benthic organisms are at risk in the areas of intensive agricultural activity (63).

Another route of exposure could be *via* inhalation through contaminated respirable dust. Higher amounts of glyphosate (11.0-19.5 µg/l) and AMPA (520-750 µg/l) were detected in the respirable dust than in the sources of emission (bulk soil and aggregate-size fractions) of agricultural soil of the central region of Argentina, after 12 months from the last application of the herbicide (22) (**Table 1**). In addition, a more recent work proposed that atmospheric deposition of glyphosate through rainfall might constitute a source of exposure of the population to this pollutant from the air, and consequently, it suggested the importance of monitor the quality of the air in Argentina (64).

The study of the toxicokinetics of glyphosate represents a crucial issue for the assessment of human health risk. The more recently work addressing this question was performed by Anadón et al. (65) in male Wistar rats. Authors reported that glyphosate was slowly and poorly absorbed through the gastrointestinal tract after oral administration of a single dose of 400 mg of glyphosate/kg; and the estimated oral bioavailability was approximately 23%. The elimination half-life of glyphosate was 14.38 h, and authors suggested that based on the apparent volume of distribution, glyphosate easily penetrated tissues (65). Other study analyzing the tissue distribution of glyphosate indicated bone, kidney, and liver as target tissues, among others (66). However, more toxicokinetic studies are needed to increase knowledge about the distribution fate of glyphosate in different tissues, organs and biological fluids, in order to predict whether glyphosate bioaccumulates in the body. Studies in rats, assessing a single oral administration of (^14^C) glyphosate, showed that this compound is poorly metabolized and that is rapidly excreted unchanged in the urine (20-30%) and feces (70-80%) (67–70). Metabolite analysis showed that AMPA was the only breakdown product detected (65, 71). Animal studies and acute ingestion cases in humans have described that glyphosate orally absorbed is metabolized to AMPA in a very low percentage (< 1%) (72, 73). The human biological half-life of glyphosate was estimated between 3 ½ and 14 ½ hours (74), indicating its rapid elimination. For this reason, urine levels of glyphosate provide a measure of recent occupational (agricultural practices and GBH manufacture) or non-occupational (aerosols, diet and drinking water) exposure to GBHs (12). Different works have detected higher levels of glyphosate in urine samples from people living near agricultural fields in comparison to those who live in urban areas (24, 75) (**Table 2**). However, other authors were unable to find differences in the levels of glyphosate or AMPA in relation to the residential environment in children and adolescents from rural regions (76). Gillezeau et al. (82), revised the literature to document human exposure to glyphosate among populations in different settings. They reported that the geometric mean for urinary glyphosate levels in occupationally exposed subjects ranged from 0.26 to 73.5 µg/l, and the environmental exposure urinary levels ranged from 0.16 to 7.6 µg/l [(for further details, see in review 82)]. On the other hand, a recent work evaluated the urinary levels of glyphosate and AMPA in Chinese workers involved in glyphosate production (i.e exposed to the herbicide at work) and detected higher concentrations of glyphosate. For glyphosate, the median was of 292 µg/l (range 20-17,202 µg/l) and for AMPA, the median was of 68 µg/l (range 10-2,730 µg/l). Furthermore, urinary concentration of glyphosate and AMPA (internal dose) was correlated with the amount of glyphosate in the air of workplace (considered as the external dose) which was <0.02 mg/m^3^–34.58 mg/m^3^ and is expressed as the time weighted average concentration of glyphosate in the air, which could reflect the actual exposure of workers (77). Despite all the above information, the number of studies reporting urinary levels of glyphosate and AMPA are still limited taking into account the extent in the usage of GBHs around the world. More studies are needed to expand the geographic regions analyzed and thus better understand the possible sources of exposure in each context.

**Table 2:** Glyphosate and AMPA concentrations in human biological samples.

Type of sample	Area	Subjects	Lab Method	LOD/LOQ		Glyphosate levels	AMPA levels	Reference
Urine	Mexico	Farmers	ELISA	LOD: 0.05 µg/l LOQ: 0.13 µg/l		0.22-0.47 µg/l	NR	(24)
Urine	USA	Pregnant women 18-39 years old	LC-MS/MS	LOD: 0.1 µg/l LOQ: 0.5 µg/l		0.5-7.2 µg/l	NR	(75)
Urine	Slovenia	Children (7-10 years old) Adolescents (12-15 years old) from rural areas	GC–MS/MS	LOQ: 0.1 µg/l		<0.39 µg/l	<0.76 µg/L	(76)
Urine	China	Workers manufacturing glyphosate	GC–MS	LOD glyphosate: 20 µg/l LOD AMPA: 10 µg/l	20-17200 µg/l		10–2730 µg/L	(77)
Pregnant women and umbilical cord serum	Thailand	Pregnant women 19-35 years old	HPLC	LOD 0.4: µg/l		Serum: 0.2-189.1 µg/l Umbilical cord: 0.2-94.9 µg/l	NR	(78)
Breast milk	USA	Lactating women 22-39 years old	ELISA	NR		3 samples ranging from 76 to 166 µg/l and 7 samples <75 µg/l	NR	(79)
Breast milk and urine	USA	Lactating women > 18 years old	HPLC-MS/MS	Milk LOD: 1 µg/l Urine LOD: 0.02 µg/l (glyphosate) and 0.03 µg/l (AMPA)		Breast Milk: ND Urine < 1.93 µg/l	Breast Milk: ND Urine < 1.33 µg/L	(80)
Breast milk	Germany	Lactating women 22-39 years old	GC-MS/MS LC-MS/MS	LOQ: 1 µg/l		Glyphosate: ND	NR	(81)

ELISA, enzyme-linked immunosorbent assay; GC, gas chromatography; HPLC, High Performance Liquid Chromatography; LC, liquid chromatography; LOD, limit of determination; LOQ, limit of quantification; MS/MS, tandem mass spectrometry; MS, mass spectrometry; ND, not detected; NR, not reported.

*In vitro* and *ex vivo* human placental perfusion experiments have shown that glyphosate is able to cross the placenta (83, 84). In addition to that, glyphosate levels have been detected not only in serum of pregnant women at childbirth (0.2-189.1 µg/l) but also in umbilical cord samples (0.2-94.9 µg/l) (78) which support the idea that glyphosate can reach the fetus (**Table 2**). Moreover, high frequency (more than 90%) pregnant women were reported to have detectable levels of glyphosate in urine residing in rural and urban areas (75). Based on this data, the herbicide glyphosate could affect the mother and child during pregnancy. Regarding glyphosate exposure through breastfeeding, a non-peer-reviewed report informed that glyphosate was present in 3 out of 10 breast milk samples ranging from 76 to 166 µg/l (79). Later, two studies found no evidence of transfer of glyphosate into milk. One of them reported that neither glyphosate nor AMPA were detected in breast milk, although lactating women had urine detectable levels of glyphosate and AMPA (80). Similar results were obtained by Steinborn et al. (81), who analyzed 114 breast milk samples from German women finding no detectable levels of glyphosate (**Table 2**). Further independent research is advisable to confirm this critical route of exposure using adequate sample size of the cohort and assessing other geographical contexts (occupational *vs.* environmental exposure).

As we have previously shown, the massive use of the herbicide and the possibility of finding it in multiple matrixes, including biological samples, led that many efforts have been invested in studying sensitive and low-time consuming methodologies to detect glyphosate and AMPA. In the last years, the most frequently used methodologies to detect these compounds were chromatography-mass spectrometry: liquid chromatography (LC) or high-performance liquid chromatography (HPLC) and gas chromatography (GC) coupled with tandem mass spectrometry (MS/MS) and enzyme-linked immunosorbent assay (ELISA). Also, other techniques of great interest such as electrochemical sensors and cell biosensor have been developed [for further details, see review (85)]. Nowadays, HPLC-MS/MS is considered the most suitable technique for the detection of phosphonic and amino acid type herbicides such as glyphosate at low concentrations (86). Furthermore, HPLC-MS/MS has higher recovery values when compared to GC-MS/MS (81). The particular physicochemical characteristics of glyphosate and AMPA (low volatility and high water solubility) make the detection of both at trace levels difficult, in addition to the need of using additional steps such as derivatization since the lack of chromophore or fluorophore groups (87). The limits of detection for the methodologies available have been decreased over time which have helped to improve the quality of data in relation to glyphosate levels to what humans and living organisms are exposed (88). For example, a recent work analyzing urine levels in Portuguese adults reported highly sensitive and low limits of detection (LOD) for glyphosate of 0.02 μg/l and for AMPA of 0.013 μg/l, with a limit of quantification (LOQ) for both of 0.05 μg/l applying HPLC-MS/MS (89). Regarding ELISA method, it has been proved to be sensitive since the LOD are better than GC-MS/MS, and even similar of those obtained by HPLC-MS/MS (90). The disadvantages of ELISA are the high limits of AMPA detection and the occurrence of false positives as a result of cross-reactivity with other organic contaminants (91). At the same time, it is considered a cost-effective method for routine analysis but commercial kits are relatively expensive (92). Researchers claim for the development of new approaches to detect glyphosate in a fast, easy and effective manner using a portable device for real time assays in the field (85). **Tables 1** and **2** include the methods utilized, LOD and LOQ for different glyphosate determinations in food, environmental and biological samples. Monitoring of these matrixes to estimate the levels of human exposure to glyphosate, as well as, networking between institutions with the appropriate platforms or equipment to detect pesticides should be encouraged by governments.

## Estrogenic Effects of Glyphosate and GBHs in Cell Culture and Animal Models

Some environmental chemicals exhibit estrogen-like properties, acting directly by activating or inhibiting estrogen action, or indirectly by modulating its action and consequently, altering the normal regulatory function of the endocrine system (93). In this sense, several *in vitro* and *in vivo* studies have been performed to elucidate whether glyphosate and/or GBH are able to induce estrogenic effects.

Regarding *in vitro* studies, potential targets involved in the estrogenic pathway were evaluated in multiple cell lines, such as human placenta (94), human embryonic kidney (HEK)293 cells (95), bovine granulosa and theca cells (33, 96), human breast cancer cells (97, 98) and human endometrial carcinoma cells (99). The enzyme aromatase cytochrome P450 which converts androgens into estrogens and also, considered a limiting step for estrogen biosynthesis was assessed (100). This enzyme is implicated in several physiologic functions, including female and male gametogenesis, sex differentiation, reproduction and bone growth (101). Richard et al. (94), demonstrated that GBH decreased aromatase activity in association with a downregulation of its gene expression in human placental JEG3 cells working with concentrations lower than those used with agricultural purposes. In the same work, both glyphosate and GBH had an inhibitory effect on aromatase activity in human placental and equine testicular microsomes and proved to interact with the active site of the purified enzyme. Similarly, Benachour et al. (95), showed that both GBH and glyphosate inhibit aromatase activity in HEK 293 cells, placental-derived JEG3 cells, and extracts from human placenta and mammalian testis. Another potential target of glyphosate and GBH is the product of aromatase enzyme, the hormone estradiol (E2). Ovarian granulose cells were evaluated as they are the main source of E2 secretion. Some authors showed that glyphosate and GBH inhibit E2 secretion in granulose cells from farm animals (cows and swines) (33, 96, 102). These results evidence that glyphosate and GBH disrupt aromatase expression and ovarian steroid production *in vitro* with potential negative consequences for the reproductive system, antagonizing estrogenic effects. Kiyama and Wada-Kiyama (93) listed in their review estrogenic chemicals that have exhibited contradictory results depending on the assay and concentration evaluated. Therefore, estrogenic compounds include chemicals that have not only estrogenic effects but also anti-estrogenic ones. Other researchers investigated estrogenic effects of glyphosate and GBHs on human breast cancer cells. Glyphosate induced the proliferation of T47D and MCF-7 cells, hormone-dependent human breast cancer cell lines; however, it failed to produce proliferation in MDA-MB231 cells, a hormone-independent human breast cancer cell (97, 98). These results allowed to suggest that estrogen receptor (ER) signaling may be involved in the glyphosate-induced proliferative effects. Later, when T47D cells were exposed to ICI 182,780 (Fulvestrant, the first steroidal ‘pure’ antioestrogens), the proliferative effects of both glyphosate and E2 were mitigated (97, 98). Additionally, glyphosate was seen to stimulate estrogen response element (ERE)-mediated transcription of a luciferase reporter gene and ICI 182,780 blocked the stimulatory effects (97, 98). Based on these results, glyphosate induce cell proliferation and ERE activation through ER. Thongprakaisanget al. (97), also found that glyphosate induces both ERs with different patterns. While ERβ activation occurs in a rapid way, ERα activation is slower but longer. Molecular dynamic simulations were used to evaluate and compare E2 and glyphosate binding energies to ERα. This analysis showed that glyphosate binds at the active site of the receptor in a weak and unstable mode, which indicates that glyphosate is unlikely to activate ERα directly (98). It is known that estrogenic effects can be caused by an activation of ER in a ligand-independent manner. So, Mesnage et al. (98), proposed that glyphosate might increase ERE-luciferase reporter gene expression by a ligand-independent mechanism which could involve an interplay with cellular signaling pathways. More recently, a work from our lab studied whether glyphosate was able to cause similar effects as E2 on epithelial-mesenchymal transition-related process by using the Ishikawa cell line (a human endometrial carcinoma cell line) (99). Glyphosate promoted cell migration and invasion, and down-regulated E-cadherin mRNA expression in a similar way to E2. All the mentioned effects were reversed after treatment with ICI 182,780. Altogether, the results suggested that glyphosate could act *via* ER-dependent pathway and most importantly, glyphosate might stimulate the estrogenic pathway in a tumoral microenvironment (99). **Table 3** summarizes the most relevant results of mechanistic evidence of glyphosate and GBH estrogenic-like properties.

**Table 3:** *In vitro* and *in vivo* assays showing estrogenic-like properties of glyphosate (Gly) and glyphosate-based herbicides (GBHs).

Compound tested	Exposure type	Estrogenic-like effects	Reference
***in vitro assays***			
Glyphosate (Gly)	**Model:** T47D cells (hormone-dependent human breast cancer cells) **Exposure:** 24 h **Concentration:** 169x10^-12^ to 169x10^-6^ g/l	-Gly induced proliferation -Gly induced estrogen response element transcriptional activity blocked by the estrogen antagonist ICI 182,780. -Gly increased the expression of ERα and ERβ -Antagonist effect of Gly in presence of estradiol (E2)	(97)
-GBHs: Roundup ProBio, Glyphogan, Roundup Grand Travaux Plus, and Roundup Original DI -Glyphosate (Gly)	**Model:** T47D and MCF-7 cells (hormone-dependent human breast cancer cells) **Exposure:** 24 h or 6 days (depend on the assay) **Concentration:** 10x10^-6^ to 1,000,000 x10^-6^ g/l	-Gly induced proliferation -Gly induced estrogen response element transcriptional activity blocked by the estrogen antagonist ICI 182,780.	(98)
Glyphosate (Gly)	**Model:** Ishikawa cells (human endometrial carcinoma cells) **Exposure:** 24 h **Concentration:** 33.8 x10^-6^ and 338 x10^-6^ g/l	-Gly induced cell migration and invasion -Gly decreased E-cadherin mRNA expression -Gly effects were reversed by the estrogen antagonist ICI 182,780	(99)
***in vivo assays***			
GBH	**Model:** adult ovariectomized female rat **Exposure:** for 3 consecutive days after ovariectomy through subcutaneous injection **Dose:** 0.5, 5 and 50 mg Gly/kg/day **Target organ:** uterus	-GBH increased luminal epithelial height -GBH decreased ERα mRNA levels -GBH disrupted estrogen-responsive gene expression	(103)
GBH: Roundup Full II	**Model:** ovariectomized female rat on postnatal day (PND)21 **Exposure:** on PND1, 3, 5, and 7 through subcutaneous injection + E2 implants from PND21 to PND60 **Dose:** 2 mg Gly/kg/day **Target organ:** uterus	-GBH increased luminal epithelial height -GBH increased E2-induced cell proliferation -GBH deregulated ERα and ERβ expression -GBH enhanced the response to E2	(104)

GBH, Glyphosate-based herbicide; Gly, glyphosate (active ingredient); E2, 17β-estradiol; ERα, estrogen receptor alpha; ERβ, estrogen receptor beta; PND, postnatal day.

Among *in vivo* studies, in our laboratory we have explored estrogenicity of GBH using different experimental approaches (103, 104). The results showing *in vivo* estrogenic-like properties are summarized in **Table 3**. Firstly, Varayoud et al. (103) evaluated the potential estrogenic effects of a GBH formulation by a classical *in vivo* test, the uterotrophic assay. We used ovariectomized rats, which were subcutaneously injected with a GBH formulation in doses of 0.5, 5, or 50 mg glyphosate/kg bw/day or with an uterotrophic (2.10^-5^g E2/kg/day) or a non-uterotrophic (2.10^-7^g E2/kg/day) dose of E2. The uterine wet weight, which is the hallmark of this assay, was not increased by GBH treatments as it was expected for an estrogenic compound. However, the herbicide altered estrogen-sensitive genes and ER protein expression in the uterus, similar to the effects of a non-uterotrophic dose of E2 (103). In a more recent work, we studied how neonatal exposure to 2 mg glyphosate/kg bw/day dose of a GBH in Wistar rats impacts on the uterus response to E2 later in life (104). Interestingly, our results showed that GBH exposure increased the sensitivity to E2 in ovariectomized rats, accompanied of histomorphological changes referenced as endometrial hyperplasia characterized by an increase of luminal epithelial height, high stromal nuclei density and proliferation (105). In addition to these morphological alterations, changes in molecules pointed out like E2-modulated targets and implicated in uterine E2 responsiveness were observed (104). Furthermore, several studies have reported deregulation of E2 levels, ERα protein and gene expression, and E2-dependent genes after GBH or glyphosate exposure in animal models (31, 35, 36, 106, 107).

The potency of estrogenic compounds is a complicated issue since the effects depend on the end point, receptor type, pathway, tissue, window of exposure, among others. As an example, Bisphenol A (BPA) sometimes is called as a “weak” estrogen because of its relatively weak binding/activation of the nuclear receptors compared to E2, although this is not always the case (108). However, when the non-genomic estrogenic activity of BPA was measured, it resulted comparable or more potent than E2 (108). In the case of Gly and GBH, *in vitro* studies show that both have estrogenic properties, which are weaker in comparison to E2 considering the present evidence. In addition, *in vivo* results show that estrogen pathway is a sensitive target of GBHs and that these herbicides might exacerbate the response to environmental estrogens. However, further *in vitro* and *in vivo* research is warranted to compare the estrogenic potency of glyphosate and its formulations in relation to E2 and other endocrine disruptor chemicals that have been defined as estrogenic compounds (such as BPA).

## Adverse Reproductive Outcomes of Glyphosate and Its Formulations

Infertility is estimated to affect up to 15% of couples worldwide (109), and remains an ongoing reproductive problem, despite advances in assisted reproductive techniques (110). Growing evidence has shown that lifestyle factors and exposure to polluting chemicals with endocrine disrupting properties represent potential risks factors associated with alterations of the reproductive health, including subfertility/infertility (111, 112). In this sense, several epidemiological studies showed an association between environmental and/or occupational exposure to pesticides and male and female infertility and adverse pregnancy outcomes (113–119). As for GBHs, although they are the most widely used pesticides worldwide, to date, human studies addressing the effects on reproductive health are scarce. A retrospective study performed in a rural population from Ontario (Canada) showed that pre-conception exposure to glyphosate was associated with elevated risk of late abortions (120). In a more recent study, higher glyphosate urine levels were correlated with shortened gestational lengths in an Indiana cohort (USA) of pregnant women from rural and non-rural areas (75). In another work from Thailand, higher levels of glyphosate were detected in pregnant women who work in agriculture or live in families that work in agriculture (78).

Although more epidemiological data are necessary, growing evidence from animal studies has shown detrimental effects of glyphosate and GBHs on reproductive health at environmentally relevant doses, including pre- and post-implantation embryo losses (31, 34, 121, 122), fetal growth retardation and structural congenital anomalies (121–123). These findings are discussed in the following sections.

### Effects on Female Reproductive Performance

Most *in vivo* studies that aimed at evaluating the effects of glyphosate and GBHs on female reproduction have been conducting using rodents as animal models. The first report showing female fertility failure by GBH exposure was published in our lab by Ingaramo et al. (34). In that work, we exposed female rats to a low dose of the herbicide, i.e. 2 mg of glyphosate/kg bw/day, which is in the order of magnitude of the currently reference dose (RfD) (1 mg of glyphosate/kg bw/day) according to EPA (124) and 4-fold higher than the ADI established by EFSA (125). GBH was administered on postnatal days (PND) 1, 3, 5 and 7, by subcutaneous injections. On PND90, females were mated with males of proven fertility to evaluate the reproductive performance on gestational day (GD) 19. We found that although all GBH-exposed females became pregnant, neonatal exposure to the herbicide significantly increased the number of resorption sites leading to an increase in the rate of post-implantation embryo losses. It is important to highlight that neither the number of corpora lutea (CLs), nor the number of implantation sites were altered. In a later study, we investigated the effects on fertility of two doses of a GBH (i.e. 2 mg/kg/day and 200 mg/kg/day) applying a different experimental approach (121, 122). In this experiment, pregnant rats (F0) received the GBH through food, which is a more representative route of human exposure, from GD9 until weaning, and the reproductive performance was evaluated in sexual matured F1 females. Similar to our previous findings, no alterations in the pregnancy rate and number of CLs were found; however, no differences were detected in the number of resorption sites. Importantly, a significant lower number of implantation sites, in association with an increase of the rate of pre-implantation embryo losses were recorded for the higher GBH dose studied. We also observed an abnormal implantation pattern in both GBH-exposed groups, characterized by unilateral pregnancies without alterations in the number of CLs from the ovary adjacent to the non-pregnant uterine horn. More recently, we performed two comparative studies led to evaluate the effects of GBH on fertility. One of them was performed to compare glyphosate alone *vs* GBH, to analyze the possible differences between commercial formulation and pure glyphosate (31). Interestingly, in this work we detected 3-fold higher serum levels of glyphosate in the GBH-treated F0 dams in comparison with Gly-treated rats. On the other hand, Panzacchi et al. (126) quantified the levels of glyphosate in urine samples from F0 dam rats and their offspring which were exposed to a GBH formulation or Gly through drinking water. They found a tendency to detect higher levels of glyphosate in GBH-exposed animals. Based on this evidence, it might be proposed that co-formulants, additives incorporated to GBH formulations, could alter the absorption and/or excretion of glyphosate increasing its levels in serum or urine. This study showed that both GBH and Gly cause an increase in the rate of pre-implantation embryo losses, suggesting that the active principle might be the main responsible for the effects observed (31). Despite the levels of glyphosate reached in serum were different between the treatments, both compounds induce similar deleterious effects. In the second study, we aimed to assess the effects of GBH in comparison with a mixture of commercial formulations of glyphosate and endosulfan (127). The pesticide mixture produced adverse reproductive effects that were similar to that induced by GBH alone, indicating a predominant effect of the herbicide (127). According to these results, we provided evidence which indicate that the deleterious effects are produced by the active ingredient glyphosate. In addition, we consider that it is necessary to increase information about the effects of mixtures of pesticides which represent more realistic scenarios to mimic the environmental exposure.

To our knowledge, only one report from other lab found adverse effects of GBH on female fertility when assessing F0 dams unlike our work in which we evaluated F1 dams (128). In that work, pregnant female rats were exposed to a sub-lethal dose of GBH alone (500 mg/kg) or in association with other herbicide (500 mg/kg of a GBH plus 50 mg/kg of Paraquat) administered by gavage during early pregnancy from GD1 to GD7, and the assessment of the reproductive capability was performed on the seventh day of pregnancy. The results showed that individual or combined exposure to herbicides decreased implantation sites and increased pre-implantation embryo losses. Authors also reported decrease in the ovary weight and in the total number of CLs (128). Although some studies have demonstrated that GBHs impaired ovarian function *in vivo* (36, 129), the consequences of these detrimental effects on fertility were not evaluated yet.

Overall, the reported effects on fertility differed between studies. Some of them are associated with increased post-implantation embryo losses (34) and others with increased pre-implantation embryo losses (31, 121, 122, 127, 128). These differential effects could be probably due to differences in the experimental approaches, such as, administration routes (subcutaneous injections vs. diet or gavage treatment), window/lengths of exposure (brief postnatal exposure vs. gestational/lactational exposure), among other factors that might impact on the physiology of the exposed organism. Despite that, all these findings provide evidence that the active principle glyphosate, GBHs, or the herbicide being part of a pesticide mixture cause adverse effects on female reproductive capability.

### Hormonal, Molecular and Epigenetic Alterations Associated With Implantation Failures

Our previous results showed that *in utero* and lactational exposure to Gly or GBH impairs female fertility by reducing the number of implanted embryos and increasing the rate of pre-implantation embryo losses (31, 121, 122). This section aims to integrate the knowledge available to date on the mechanisms that might explain the implantation failures, with focus on hormonal, molecular and epigenetic alterations, which might affect the uterine receptivity.

Embryo implantation is a complex process that requires the temporal interaction between developmentally competent blastocysts and a receptive uterus (130). Failure of the embryo to implant is a major cause of infertility, and is mainly associated with impaired uterine preparation to achieve the receptive stage (131). Uterine receptivity refers to the window of limited time in which the endometrium undergoes morphological, cellular and molecular changes conducive to blastocyst attachment, and subsequent pregnancy establishment (132). The set of these complex uterine changes, known as functional differentiation, is orchestrated by the ovarian hormones estrogen and progesterone. These hormones act primary *via* their nuclear receptors, progesterone receptor (isoforms PR-A and PR-B) and estrogen receptor alpha (ERα), to direct transcriptional pathways in the endometrium leading to the establishment of the “window of receptivity”, i.e. the period in which the uterus is permissive to embryo implantation (132). Many hormone-regulated signaling pathways are involved in uterine receptivity; some of them include transcription factors, growth factors, cytokines, and diverse signaling molecules (133, 134).

In recent years, our work was focused on elucidating the mechanisms responsible for the implantation failures by Gly and GBH exposure. For that purpose, F1 females which were perinatally exposed to a low dose (2 mg/kg/day) of Gly or GBH, became pregnant and hormonal and uterine targets were evaluated on GD5 (during the pre-implantation period). Higher serum levels of E2 along with increased uterine ERα expression were detected in Gly- and GBH-exposed females (31). It is well known that estrogen levels are critical in determining the window length of uterine receptivity for blastocyst implantation (135, 136). Low estrogen levels tend to extend the period of receptivity, while higher serum levels and/or ERα expression may close the window in advance, leading the uterus to a refractory state (135). Considering this information, our findings suggest that hormonal imbalance prompted by Gly and GBH might short the window of receptivity which lead to the decreased implantation rate.

As previously stated, several *in vitro* and *in vivo* studies have found ovarian dysfunction by Gly or GBH treatment. Some of the deleterious effects include: altered ovarian morphology, impaired folliculogenesis, disruption of aromatase activity (enzyme responsible for estrogen synthesis), hormonal imbalance, and increased oxidative stress, among others (33, 36, 94, 96, 102, 129, 137). It is worthy to mention that although we did not assess the ovarian function, the ovulation rate and the CL “activation” were conserved in females perinatally exposed to Gly or GBH, as no changes were detected in the number of CLs (31, 121, 122). In accordance with these results, it has been reported that the sustained increase of E2 during the follicular phase did not affect oocyte and embryo quality but had detrimental effect on implantation and pregnancy (138).

To deeply understand the mechanisms involved in Gly- and GBH-induced implantation failures, we analyzed the expression of hormone-responsive genes, which are essential for successful implantation. Two genes, the Homeobox A10 (*Hoxa10*) and the Leukemia inhibitory factor (*Lif*), were downregulated in the pre-implantation uterus of females exposed to Gly and GBH (31). *Hoxa10* is a transcription factor that plays a dual role; during embryogenesis *Hoxa10* drives the development and patterning of the uterus, and at adulthood regulates uterine receptivity, implantation and subsequently endometrial decidualization (139). On the other hand, *Lif* is a pro-inflammatory cytokine belonging to the interleukin-6 family also required for implantation, which participate in both endometrial preparation and embryo attachment (140, 141). In this sense, gene targeting studies performed in mice have contributed to shed light on the importance of these genes for the success of the implantation process. It has been demonstrated that targeted mutation of either *Hoxa10* or *Lif* in mice did not impair embryo viability but alter uterine differentiation at the receptive stage leading to implantation failures. Interestingly, embryos from *Hoxa10* or *Lif* knockout females normally implant when transferred to wild-type surrogates, indicating that maternal uterine alterations are responsible for such effects (142–146). Under physiological conditions, maximal expression of both *Hoxa10* and *Lif* occurs at the window of receptivity, which coincides with a peak in the E2 serum levels (139, 146). It was reported that although estrogen at physiological range stimulates *Lif* expression, an abnormal increase of this hormone induces an inhibition of *Lif* leading to defective implantation (147). These findings are in accordance with our results showing significantly higher E2 serum levels along with lower expression of *Lif* in the uterus of Gly and GBH-exposed females exhibiting lower implantation rates (31). Human clinical evidence further supports the requirement of *Hoxa10* and *Lif* for uterine receptivity, based on the association between defective expression of both genes and recurrent implantation failures, infertility, and gynecological disorders including endometriosis, and polycystic ovary syndrome in women (148–152).

Some studies have evaluated the action of glyphosate or its formulations as possible endocrine disruptors. Many of them have found, as common outcomes, alterations in i) the E2 biosynthesis, ii) the levels of ER expression, iii) the signaling pathways or cellular events that are under the control of estrogens (98, 102, 104). As we mention before, timely and normal E2 serum levels and/or ERα expression are crucial in determining the window of uterine receptivity and therefore the success of implantation. Based on this background, we proposed that alterations of ERα gene expression could explain, at least in part, the effects of GBH on implantation. So that, we investigated whether the levels of expression of uterine ERα gene were altered, and whether epigenetic mechanisms could be involved in the disruption of implantation events (37). Previous reports have been proved that environmental exposure to polluting chemicals prompt epigenetic marks such as, DNA methylation, PTMs, and noncoding RNAs, leading to changes in gene transcription, and even to transgenerational inheritance of such epigenetic alterations (153). In our work, we found that perinatal exposure to GBH induces long-term epigenetic modifications in the O promoter of ERα rat gene characterized by DNA hypomethylation, and alterations in PTMs (increase of histone H4 acetylation and histone H3 lysine 9 trimethylation (H3K9me3), and decrease of H3K27me3 (37). Altered methylation/acetylation pattern of ERα promoter regions have been found in different pathological conditions such as breast and colon cancer, uterine leiomyomas, and ovarian endometrioma (154–157). Our findings show that epigenetic disruption of the uterine ERα gene could be linked to the GBH-induced implantation failures.

In the last years, there has been growing evidence showing that glyphosate and GBH are able to cause epigenetic alterations in cell-culture assays and *in vivo* animal studies. Some of the epigenetic modifications reported include decrease of global DNA methylation and altered methylation pattern of tumor suppressor genes in peripheral blood mononuclear cells (158, 159), differential methylated DNA regions in rat sperm (38), changes in the methylation status of Erα promoters in rat mammary gland (107), and finally, differential expression of non-coding RNAs in mouse brain (160, 161). Importantly, these epigenetic changes have been involved in diverse pathological processes, such as cancer development (162, 163), reproductive disorders (164, 165), and neurodegenerative diseases (166).

### Adverse Effects on Health of Successive Generations

Maternal exposure to environmental chemicals during the pre- or perinatal period is considered an extremely sensitive window of exposure, which might affect embryogenesis or organogenesis leading to multiple types of defects in the progeny (167, 168). The consequences are evidenced by morphological, functional or biochemical alterations which in turn, lead to stillbirth, physical or cognitive disabilities or could predispose to certain pathologies at long-term (169–171). Evidences over the years have shown that these effects are not limited to the first generation (F1) but also may be transmitted to a second (F2) or even successive generations (172). An issue of particular concern regarding glyphosate and its formulations is the effect on offspring and subsequent generations in both animals and humans. In this section, we will address the effects on health of successive generations after maternal exposure to glyphosate or GBHs, even though paternal exposure is also an important factor. **Table 4** summarizes the effects reported in F1 and F2 offspring in animal models after glyphosate or GBH exposure and the experimental conditions of the studies discussed in the present section.

**Table 4:** Effects of glyphosate (active ingredient) (Gly) and glyphosate-based herbicides (GBHs) associated with female fertility and those reported in the successive generations (F1, F2 or F3) of mammalian offspring after maternal exposure.

Compound tested	Exposure type	Fertility associated effects	F1, F2 or F3 offspring effects	Reference
-GBH: Magnum Super II -Gly	**Model:** pregnant female rat **Exposure:** trough food from gestational day (GD)9 to lactational day (LD)21 **Dose:** 2 mg Gly/kg bw/day **Target of study:** uterus	**F1 female rats** ***GD5:*** **Gly and GBH:** -increased E2 serum levels -altered expression of implantation related- molecules: (ERα, Hoxa10 and Lif*)* ***GD19:*** **Gly and GBH:** *-*increased rate of preimplantation losses	**Gly:** -decreased weight of F1 male pups at birth	(31)
-GBH: Roundup Full II	**Model:** pregnant female rat **Exposure:** subcutaneous injections from postnatal day (PND)1 to PND7 **Dose:** 2 mg Gly/kg bw/day **Target of study:** uterus	***GD9:*** -altered endometrial decidualization process -increased proliferation (Ki67) ***GD19:*** -increased rate of postimplantation losses	NR	(34)
-GBH: Roundup -Gly	**Model:** pregnant female mouse **Exposure:** trough water from GD1 to GD19 **Concentration**: 5 mg Gly/ml **Target of study:** ovary, hypothalamus and pituitary gland.	***GD19:*** **Gly and GBH:** -decreased ovary weight and altered ovarian histology. -decreased progesterone serum levels -altered expression of genes involved in hormonal balance in hypothalamus, pituitary gland and ovary: GnRH, LHR and3β-HSD -altered serum and ovarian markers of oxidative response **Gly:** -increased estradiol serum levels **Gly or GBH** -specific treatment-related differences	**Gly:** -altered sex ratio of F1 fetuses (increased male to female ratio)	(36)
-GBH: Magnum Super II	**Model:** pregnant female rat **Exposure:** through food from GD9 to LD21. **Dose:** 350 mg Gly/kg bw/day **Target of study:** uterus	**F1 female rats** ***GD5:*** -increased ERα mRNA expression by increasing ERα-O transcript variant -Epigenetic alterations in the O promoter of ERα	NR	(37)
-Gly	**Model:** pregnant female rat (F0) **Exposure:** trough daily intraperitoneal (ip) injections from GD8 to GD14 **Dose:** 25 mg Gly/kg bw/day **Target of study:** multiple organs	-no changes in fertility rates	-decreased body weight at weaning in F1 offspring -increased age at puberty for F1 and F2 generations -increased obesity in F2 and F3 generations -increased frequency of histological alterations in F1, F2 and F3 generations (ovary, kidney, testis, prostate) -parturition abnormalities in F2 and F3 dams -increased tumor development in F2 generation (mammary adenomas) -differential methylation genes in sperm from F1, F2 and F3 generations	(38)
-GBH: Magnum Super II	**Model:** pregnant female rat (F0) **Exposure:** through food from GD9 to LD21. **Dose:** 2 and 200 mg Gly/kg bw/day **Target of study:** uterus, placenta, fetal parameters	**F1 female rats** ***GD19:*** **200 mg Gly/kg bw/day** *-*increased rate of preimplantation losses	**F2 offspring** ***GD19:*** **200** mg Gly/kg bw/day:** -decreased fetal weight and length -increased placental index -structural congenital anomalies **2 mg Gly/kg bw/day:** **-**decreased fetal weight	(121, 122)
-GBH: Roundup	**Model:** pregnant female rat **Exposure:** by gavage from GD6 to GD15 **Dose**: 500, 750 and 1000 mg Gly/kg bw/day **Target of study:** multiple organs	-no changes associated with female fertility	**All doses:** -increased occurrence of delayed ossification in several body structures in F1 fetuses	(123)
-GBH: Roundup Full II -Mixture (MIX): commercial formulations of Gly + endosulfan (Endo)	**Model:** neonatal and pregnant female rat **Exposure:** subcutaneous injections from PND1 to PND7 **Dose:** 2 mg Gly/kg bw/day or 2 mg Gly/kg bw/day + 600 µg Endo/kg bw/day **Target of study:** uterus	***PND8*** *:* **GBH and MIX:** -increased incidence of luminal epithelial hyperplasia -increased PR and Hoxa10 protein expression ***GD19:*** **GBH and MIX:** **-**increased rate of postimplantation losses.	NR	(127)
-GBH: Roundup -Mixture (MIX): commercial formulations of Gly + Paraquat (Pq)	**Model:** pregnant female rat **Exposure:** subcutaneous injections from GD1 to GD7 **Dose:** 500 mg Gly/kg bw/day or 500 mg Gly/kg bw/day + 50 mg Pq/kg bw/day **Target of study:** uterus, ovary and embryonic cells	***GD7:*** **GBH and MIX:** **-**decreased body and ovary weight -decreased number of corpora lutea -increased rate of preimplantation losses -decreased surface and glandular epithelia and diameter of endometrial glands **MIX:** -disorganization of the cytotrophoblast and cell degeneration within the blastocyte cavity	NR	(128)
-GBH: Kalach 360 SL	**Model:** adult female rat **Exposure:** trough water during 60 days **Dose:** 126 and 315 mg Gly/kg bw/day **Target of study:** ovary	***At the end of the treatment:*** -impaired folliculogenesis and ovary development. -necrosis, vacuolisation of follicles, dissociated oocytes and granulosa cells. -increased atretic follicles. -decreased E2 serum levels -decreased antioxidant enzyme activities: catalase (CAT), superoxide dismutase and glutathione peroxidase (GP).	NR	(129)
-GBH: Roundup Bioflow -Gly	**Model:** pregnant female rat **Exposure:** through drinking water from GD6 to 6-weeks or 13 weeks after weaning **Dose:** 1.75 mg Gly/kg bw/day **Target of study:** multiple targets	NR	**F1 offspring** **Gly and GBH:** -altered developmental parameters (anogenital distance, age at first estrous) -altered sexual hormone concentrations -specific sex-related and treatment-related differences	(173)
-GBH: Roundup-Gly	**Model:** pregnant female mouse **Exposure:** trough drinking water from GD1 to GD21 **Dose**: 5 mg Gly/ml **Target of study:** liver	NR	**F1 offspring** **Gly and GBH:** -decreased body weight at weaning -disruption of lipid metabolism	(174)
-GBH: Glifloglex	**Model:** pregnant female rat **Exposure:** trough drinking water from GD1 to LD21 **Dose:** 100 and 200 mg Gly/kg bw/day **Target of study:** brain	NR	**F1 offspring** -early onset of cliff aversion reflex and auditory canal opening. -decreased locomotor activity and anxiety levels -exacerbated emotionality	(175)
-GBH: Glifloglex	**Model:** pregnant female rat **Exposure:** trough drinking water from GD1 to LD21 **Dose:** 100 and 200 mg Gly/kg bw/day **Target of study:** brain	NR	**F1 offspring** -altered antioxidant status -altered enzymes activity involved in glutamatergic and cholinergic systems -impairment in recognition memory	(176)
-GBH: Roundup	**Model:** pregnant female rat **Exposure:** trough oral gavage from GD0 to LD21 **Dose**: 250 and 500 mg Gly/kg bw/day **Target of study:** brain	**-**decreased fertility rate and gestational index	**F1 offspring** -decreased number of pups per litter -decreased body weight on PND15 and PND21 Offspring: delayed reflexes and altered motor development Adults: decreased locomotor activity, sociability, learning and impaired memory	(177)
-Gly	**Model:** pregnant female rat **Exposure:** ip injections every 48 hs from GD8 to GD20 **Dose:** 24 and 35 mg Gly/kg bw/day **Target of study:** brain	NR	**F1 offspring** -decreased body weight in pups from PND19 -altered dose-dependent reflexes development, motor activity and cognitive function -altered Wnt5a-CaMKII pathway in fetal hippocampus	(178)
-GBH: Roundup Transorb	**Model:** pregnant female rat **Exposure:** by oral gavage from GD18 to LD5 **Dose:** 5 and 50 mg Gly/kg bw/day **Target of study:** brain (cerebellum and cortex)	NR	**F1 male offspring (PND90)** -altered gene expression involved in oxidant defense, inflammation and lipid metabolism -correlation of changes in gene expression with serum concentrations of oxidative stress-related metabolites	(179)
-GBH: Roundup Maxload	**Model:** pregnant female mouse **Exposure:** trough drinking water from GD5 to LD21 **Dose**: 12.5 mg Gly/kg bw/day **Target of study:** brain (prefrontal cortex, hippocampus and striatum)	NR	**F1 juvenile offspring (PND28-PND35)** -autism spectrum disorder (ASD)-like behavioral abnormalities (cognitive and social interaction deficits) -increased soluble epoxide hydrolase (sEH) expression -ASD-like behaviors prevented by oral administration of an sEH inhibitor -abnormal composition of gut microbiota	(180)

3β-HSD, 3β-hydroxysteroid dehydrogenase; ASD, autism spectrum disorder; CaMKII, Ca2+/calmodulin-dependent protein kinase; CAT, catalase; Endo, endosulfan; ERα, estrogen receptor alpha; GBH, glyphosate based-herbicide; GD, gestational day; Gly, glyphosate (active ingredient); GP, glutathione peroxidase; Hoxa10, Homeobox A10; ip, intraperitoneal; LD, lactational day; Lif, Leukemia inhibitory factor; LHR, luteinizing hormone receptor; MIX, mixture; NR, Not reported; PND, postnatal day; PR, progesterone receptor; Pq, paraquat; sEH, soluble epoxide hydrolase; Wnt5a, wingless-type MMTV integration site.

Adverse reproductive effects of glyphosate and its formulations have been explored on offspring. Some of these effects include for example altered characteristics of the fetuses or newborn offspring such as weight, length, placental index, congenital anomalies as well as, neurodevelopmental changes detected in young offspring, among others which were determined not only in F1 but also F2 offspring. Particularly, our research group have evaluated the effect of perinatal exposure to a GBH (in a dose of 2 and 200 mg/kg/day) and glyphosate (alone) (in a dose of 2 mg/kg bw/day) administered orally through food in Wistar rats from GD9 until birth and during the whole lactation period (as described above). When analyzing F1 offspring morphometric features such as weight, firstly we did not detect adverse effects in female at birth or weaning neither in Gly- nor GBH-treated group (31, 121). These results were supported by other authors´ observations comparing the effects of glyphosate and a GBH after gestational (from GD6 onwards) plus lactational exposure in Sprague Dawley rats using 1.75 mg of glyphosate/kg/day through drinking water (173). Secondly, an interesting result we obtained was a lower litter birth weight in Gly-exposed group in comparison to control at the expense of a decrease in male weight which deserve further study. Meanwhile, outbred mouse pups prenatally exposed to glyphosate or a GBH whose mother received a solution of 0.5% of the active ingredient in the drinking water during the 21 days of pregnancy showed a decreased body weight at weaning (174). Authors exposed that this effect could be explained by previous results obtained using the same experimental model where they detected a decrease in the body weight of mothers during pregnancy (36). They hypothesized that it was as a result of energy consumption derived to detoxification instead of promoting offspring growth. Other study performed in Sprague Dawley rats by Kubsad et al. (38), also found lower weight in F1 offspring at weaning after glyphosate exposure. In this work, glyphosate was administered by daily intraperitoneal (ip) injections from GD8 until GD14 in a dose of 25 mg/kg/day. The discrepancy in the results between these works could be due to the different experimental conditions used (doses, mode of administration, animal model or time of exposure).

The occurrence of adverse effects in the second-generation of pups after GBH exposure was first reported by our research group (121, 122). F2 fetuses whose mothers had been exposed *in utero* and during lactation to a GBH formulation (2 or 200 mg of glyphosate/kg bw/day) exhibited altered morphometric parameters on GD19. The higher dose induced a decrease in the body weight and length, while the lower dose caused a decrease in the fetal weight and a trend to lower fetal length; which indicates that the GBH formulation affected fetal growth. In a multigenerational animal model, gestational overexposure to dexamethasone caused similar effects on weight of F2 fetuses (181). It is known that fetal growth is a predictor of child health because its impairment may be associated with poor neurodevelopment (182) and chronic diseases later in life such as obesity, insulin resistance and type 2 diabetes, coronary heart disease, hypertension, among others  (183–185). Epidemiological reports on fetal growth are scarce given that most studies used birth weight as a proxy measure of intrauterine growth (186). Importantly, several epidemiological studies have found a significant association between environmental or occupational maternal exposure to pesticides and decreased birth weight and length (187–190).

In addition to the effect on fetal parameters, placental index which is defined as the ratio of placental weight to fetal body weight was found to be high in F2 offspring from dams exposed to GBH in a dose of 200 mg of glyphosate/kg bw/day. Our results indicated that the higher placental index was at expense of a decrease in fetal weight (122). The placental index reflects the balance between fetal and placental growth. In normal conditions, placental index decreases across gestation as the placenta matures and the fetal weight increases (191). It allows to estimate how placental development adapts to reach fetal nutritional requirements (192). It has been suggested that adaptations take place in order to maintain appropriate fetal growth, and a failure in this process may result in a fetus that is either small or large with respect to its genetic growth potential (193). According to Macdonald et al. (194), obstetric outcomes, particularly indicators of fetal hypoxia and placental dysfunction, were associated with elevated placental index, which might explain our experiment results. Moreover, placental index may evidence maternal diseases and predict pregnancy outcomes, perinatal morbidity and mortality, and child growth and development (191, 195, 196). In this regard, a retrospective study of 18,386 pregnancies found a high placental index associated with pregnancies characterized by poor outcomes, such as hypertensive disorders, impaired fetal growth and complicated post-natal infant care (197). So far, the mechanisms leading to the alterations of these feto-placental parameters induced by perinatal exposure to GBH remains to be elucidated.

**Figure 1:**
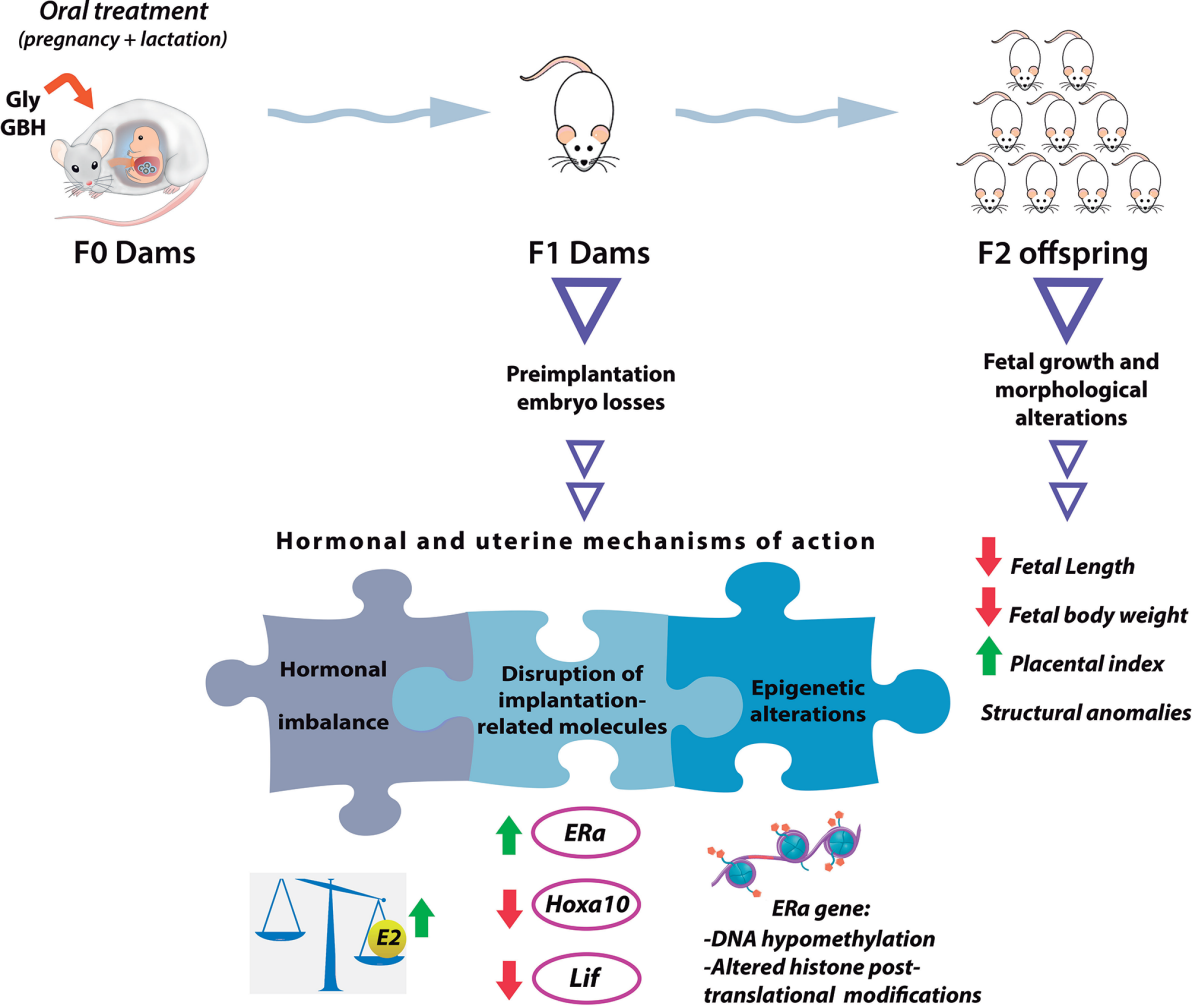
The scheme summarizes the multigenerational effects of the herbicide glyphosate (active ingredient and a glyphosate-based herbicide) on F1 adult female rats and their F2 offspring after perinatal exposure (during gestation and lactation) through food. The color green or red of the arrows denotes induction or inhibition of the molecular targets, respectively. Gly, glyphosate (active ingredient); GBH, glyphosate-based herbicide; E2, 17β-estradiol; ERα, estrogen receptor alpha; Lif, Leukemia inhibitory factor; Hoxa10, Homeobox A10.

Structural congenital anomalies are other adverse pregnancy outcomes we detected in F2 offspring from mothers perinatally exposed to a GBH formulation in a dose of 200 mg of glyphosate/kg bw/day. These anomalies involved conjoined fetuses and abnormally developed limbs (i.e., fetuses lacking one of their extremities or displaying longitudinal reduction of the tail) (121, 122). The finding of congenital anomalies in F2 offspring is of remarkable importance since this kind of effects result from F1 fetal germ cells (represented by F2 generation) modification, and open the possibility to transmit these alterations to the subsequent generations through genetic or epigenetic mechanisms (198). It is worthy to mention that teratogenic potential of both glyphosate and GBHs has been shown in different species of vertebrates (zebrafish, amphibian, chicken and rat) after developmental exposure, and when testing doses of environmental relevance or below regulatory limits (for maternal and fetal toxicity) (123, 199–201). For example, a study carried out in Wistar rats observed significantly higher occurrence of delayed ossification in several body structures in F1 fetuses whose mothers were treated with a GBH formulation. At the lowest dose evaluated (500 mg of glyphosate/kg/day) which corresponds to the half of no observed adverse effect level (NOAEL) for maternal and developmental toxicity in rats (202), 33.1% of the fetuses exhibited skeletal alterations, denoting detrimental effects at doses considered to be safe. In **Figure 1**, we summarize the multigenerational effects we detected after perinatal exposure (during gestation and lactation) to a glyphosate-based herbicide and the active ingredient on F1 female rats at adulthood and their F2 offspring.

Regarding the effects on nervous system functions, agrochemical exposure has been proposed as a risk factor for human neurodegenerative disorders (203–205) and glyphosate and GBHs are not the exception. In this sense, two case reports of acute and chronic exposure to GBHs have been associated with parkinsonism, a condition similar to Parkinson’s disease (206, 207). In addition to that, a population-based case-control study in USA showed that autism spectrum disorder (ASD), a developmental disorder, was associated with prenatal exposure to glyphosate (during pregnancy) with odds ratio (OR): 1.16 (CI 1.06 to 1.27) raising by about 30% for ASD with intellectual disability (208). For cases with ASD with intellectual disability, when coadjusting for developmental period-specific exposures such as, the first year of life, the associations became strongest (OR: 1.60; CI 1.09 to 2.34). Moreover, neurotoxic effects of this herbicide have been extensively documented in experimental studies (209–211). We focused our attention on those studies reporting brain alterations in offspring from dams treated with the herbicide. For instance, Gallegos et al. (175, 176), assessed the neurobehavioral effects of a GBH formulation supplied to Wistar rats during the whole gestation and lactation period through drinking water. Researchers evaluated two doses of glyphosate of 100 and 200 mg/kg/day and offspring were subjected to a series of tests on postnatal day 45 and 90. They found a decrease in locomotor activity and anxiety, exacerbated emotionality (175) and impairment in recognition memory (176) in the exposed offspring. Later, other works evaluating a similar exposure window (gestation or/and lactation period) found that either a GBH formulation or Gly (active ingredient) induces numerous behavioral and cognitive alterations in pups and adult offspring, which is in line with previous findings (177, 178) (further details in **Table 4**). Also, the study of de Souza et al. (179), indicated that maternal GBH exposure in doses of 5 and 50 mg of glyphosate/kg/day could have lasting effects on brain functions of rat offspring. Authors reported altered expression of molecules participating in the oxidative and inflammatory response in the cerebellum and cortex of 90-day-old male rat offspring. Additionally, gene expression deregulation induced by GBH was correlated with changes in the serum concentrations of some oxidative stress-related metabolites such as, lysophosphatidylcholine and phosphatidylcholine which were associated with neurodegenerative diseases. These alterations might contribute to neural damage increasing the risk of developing neurological pathologies. Recently, a work detected ASD-like behavioral abnormalities in juvenile mouse offspring after maternal exposure to a GBH formulation. The herbicide formulation was provided to pregnant mice from GD5 until weaning in a dose of 12.5 mg/kg/day through drinking water. An increased protein expression of soluble epoxide hydrolase (sEH) was reported in the prefrontal cortex, hippocampus and striatum from juvenile GBH-exposed offspring. Interestingly, when a sEH inhibitor was supplemented during the treatment, the behavioral disturbances were prevented. So, authors proposed sEH as a factor implicated in the development of this behavior disorder (180). Also, in this work, abnormal composition of gut microbiota was detected in GBH-exposed offspring. As recent research pointed an interaction between altered microbiota and ASD (212), more studies are needed in order to explore the role of gut microbiota on glyphosate-induced ASD. To our knowledge, glyphosate- or GBH-induced brain-related disturbances have not been reported beyond the first generation of offspring. According to the current data, pre- and early postnatal exposure to glyphosate or GBH might lead to impairment of the cognitive performance and behavioral functions of offspring.

Finally, Kubsad and colleagues (38) determined that glyphosate in a dose of 25 mg/kg bw/day is able to induce transgenerational effects. They investigated a transient glyphosate exposure on gestating F0 outbred Sprague Dawley rats which were treated with daily ip injections from GD8 until GD14. Researchers evaluated the frequency of several pathologies in different generations: F0 adult females directly exposed to glyphosate, F1 and F2 offspring directly exposed in their condition as fetus and fetus´ germline respectively, and F3 offspring, the first unexposed generation to the herbicide. Alarming results indicated minor effects on F0 and F1 generations, and higher incidence of histological abnormalities in different organs (prostate, testis, kidney and ovary) and pathologies such as obesity, parturition complications and tumor development (specifically mammary adenomas) in F2 and F3 one-year old rats. Also, differential DNA methylation regions (DMRs) were identified in sperm from control and Gly-treated animals in F1, F2 and F3 generations. The most frequent DMR associated gene categories were transcription, signaling, metabolism, receptors, and cytoskeleton which are mainly involved in metabolic, signaling, cancer and endocytosis pathways (38). Although the functional role of altered DNA methylation along the generations remains to be elucidated, authors proposed that the pathologies observed in the F3 generation emerge in part, as the result of epigenetic alterations or “epimutations”. This particular phenomenon, in which F3 animals that never had contact with the chemical exhibit higher number of alterations than the previous generations, was also reported for the herbicide atrazine (213). To sum up, all the evidence presented in this section highlights the importance and need of further evaluating the toxicology of glyphosate and its formulations through the successive generations.

## Limitations of Animal Studies for Predicting Human Exposure to GBHs

A concern question about using laboratory animals as models to predict human health risk, is whether they are truly representative of actual human or environmental exposure. As stated Hartung (214) in his revision, no model is perfect, and the term ‘‘model’’ implies deviation from reality. Despite that, animal models are fundamental tools in the life sciences, and conclusions should be interpreted with caution when predicting chemical hazards or health risk. In the particular case of glyphosate, major limitations in some studies keep animals models away from representative chronic human exposure, such as, unrealistic high-dose treatment and short exposure duration, administration routes which not represent that of human exposure like subcutaneous or ip injections, toxicological assessment based on the effects of pure glyphosate instead of GBHs which can underestimate toxicity. However, in the last few years several studies have been conducted applying more complex experimental designs in an attempt to better reflect human and environmental exposure, i.e., oral exposure to the herbicide through adding it into drinking water or food, longer exposure periods (for example, during gestation and lactation), assessment of doses representative of glyphosate residues from sprayed crops (plants or seeds), or doses that are in the order of magnitude of the levels detected in different environmental matrixes, or that reflect the RfD established by regulatory agencies. However, no study replicates actual modes of ingestion, and experimental works providing animals with food contaminated with glyphosate at the levels found in food stuff or drinking water/groundwater should be performed not only in the common laboratory animal models but also, in farm animals for human consumption. To determine the actual risk of glyphosate and to help set safe exposure limits, studies are needed to determine how much glyphosate from food is taken to the body. In addition, to bridge between animal models and human exposure, dietary or environmental (air) exposure needs to be correlated with levels of glyphosate and AMPA in the body.

Another matter to be considered is the lack of studies dealing with the consequences for health of the co-formulants classified as “inert” ingredients in herbicide formulations. As co-formulants are treated as a trade secret by the manufacturers, the composition of practically most of the GBHs are unknown. Moreover, as the composition of co-formulants and their relative concentrations may differ among GBH brands, it is difficult to evaluate their contribution to the herbicide toxicity (215).

## Concluding Remarks and Future Directions

As Argentina is one of the countries with the highest consumption of glyphosate-tolerant seeds and glyphosate formulations, we know from very close experience the tight dependence of the economy on extensive agriculture and GBHs. As long as new biotechnological events which confer tolerance to glyphosate got approval, the usage of GBH formulations will continue rising. This, in addition to the fact that the yield of genetically modified crops is highly superior than those crops not modified, motivates farmers to choose the technological package of genetically modified seeds and glyphosate herbicide. Also, despite the spread of weed resistance to glyphosate, there are few initiatives to analyze strategies for farming with limited or without glyphosate use. Some alternative methodologies include precision agricultural systems, mechanical weed control systems, bioherbicides, among others (216, 217). Unfortunately, currently available herbicides, which might be proposed as alternatives to glyphosate, are very limited, less effective, and more expensive, or even may have a worse ecotoxicological profile than glyphosate (218). So, in this scenario, our role as scientists is to show the evidence to the scientific community but also, to make know what is reported in the literature to the general community. Through this way, we will be able to dialogue, educate and raise awareness on the risks of excessive use of these pesticides mainly of those who manipulate the herbicide formulations or are responsible directly or indirectly of their use. Also, it is important that people take into account potential acute effects, but also long-term effects and possible impact on the health of subsequent generations; emphasizing on the health care of women preconceptionally and during pregnancy (especially those who could be occupationally exposed to higher levels than general population). Although there is extensive accumulated experimental evidence about the negative impact of glyphosate and GBHs on pregnancy outcomes, there are just a few epidemiological studies on reproductive health to arrive to conclusive definitions. Therefore, it is urged that more assessments will be a priority at this stage and we are focusing our efforts on this issue.

## Author Contributions 

All authors contributed to the manuscript design, writing, and editing. MM, MD, and JV are Career Investigators of the CONICET. VL and MR are fellows of CONICET. All authors contributed to the article and approved the submitted version.

## Conflict of Interest

The authors declare that the research was conducted in the absence of any commercial or financial relationships that could be construed as a potential conflict of interest.

## Abbreviations

ADI, acceptable daily intake; AMPA, aminomethylphosphonic acid; ASD, autism spectrum disorder; CLs, corpora lutea; DMRs, DNA methylation regions; E2, 17β-estradiol; EFSA, European Food Safety Authority; EPSPS, 5-enolpyruvylshikimate-3-phosphate synthase; ERα, estrogen receptor alpha; ERE, estrogen response element; ERs, estrogen receptors; GBHs, glyphosate-based herbicides; GD, gestational day; Gly, glyphosate (active ingredient); H3K9me3, histone H3 lysine 9 trimethylation; HEK, human embryonic kidney; *Hoxa10*, Homeobox A10; ip, intraperitoneal; *Lif*, Leukemia inhibitory factor; MRLs, maximum residue limits; OR, odds ratio; PND, postnatal day; PTMs, histone post-translational modifications; RfD, reference dose.
